# Re-Clustering and Profiling of Digestive System Tumors According to Microenvironment Components

**DOI:** 10.3389/fonc.2020.607742

**Published:** 2021-02-10

**Authors:** Yongwei Wang, Sen Guo, Zhihong Chen, Bing Bai, Shuo Wang, Yaxian Gao

**Affiliations:** ^1^ Department of Anatomy, Basic Medical Institute, Chengde Medical College, Chengde, China; ^2^ Department of Immunology, Basic Medical Institute, Chengde Medical College, Chengde, China

**Keywords:** The Cancer Genome Atlas (TCGA), digestive system tumors, immune, stromal, microenvironment

## Abstract

**Background:**

Immunotherapy has become the most promising therapy in digestive system tumors besides conventional chemotherapy and radiotherapy. But only a few patients can benefit from different types of immunotherapies, such as immune checkpoint blockade (ICB). To identify these ICB-susceptible patients, methods are urgently needed to screen and profile subgroups of patients with different responsiveness to ICB.

**Methods:**

This study carried out analysis on patients with digestive system tumors that were obtained from Cancer Genome Atlas (TCGA) cohorts. The analyses were mainly performed using GraphPad Prism 7 and R language.

**Results:**

We have quantified the microenvironmental components of eight digestive system tumor patients in TCGA cohorts and evaluated their clinical value. We re-clustered patients based on their microenvironment composition and divided these patients into six clusters. The differences between these six clusters were profiled, including survival conditions, enriched biological processes, genomic mutations, and microenvironment traits. Cluster 3 was the most immune-related cluster, exhibiting a high infiltration of non-tumor components and poor survival status, along with an inhibitory immune status, and we found that patients with high stromal score indicated a poor response in ICB cohort.

**Conclusions:**

Our research provides a new strategy based on the microenvironment components for the reclassification of digestive system tumors, which could provide guidance for prognosis judgment and treatment response prediction like ICB.

## Introduction

Digestive system tumors are the most common tumor type and are associated with rapid malignant progression (1). Even after patients receive standard radiotherapy and chemotherapy treatment, the prognosis remains poor (2, 3). This unsatisfactory prognosis is in part due to the hidden nature of digestive system tumors, making them difficult to detect early. These tumors are often found at advanced and malignant stages, where symptoms are obvious. But the metastatic and recurrent traits of digestive system tumors make them difficult for conventional treatment programs to handle (4–8).

In recent years, more and more research has focused on the importance of the tumor microenvironment in driving malignancy, including in digestive system tumors (9–12). Most of the previous studies have only focused on tumor cells themselves and their internal mechanisms, but mutual communication and regulation exist between tumor cells and other components of their microenvironment (13–15). Through paracrine mechanisms, tumor cells could reprogram their surrounding immune and stromal microenvironments into a “pro-tumor” microenvironment. The reprogrammed microenvironment could facilitate the malignant phenotype of tumor cells, such as proliferation, invasion, migration, and pro-vasculogenic effects (16). Meanwhile, increasing evidence suggested that the disorganized microenvironment may contribute to tumor cells’ abilities to escape the effects of conventional treatments, such as chemotherapy, radiotherapy, and anti-vasculogenic therapy, as well as some classical molecular targeting therapies (17–19).

In recent years, studies have begun to focus on immunotherapy, which is considered as a promising and upcoming therapy that has been extensively used in basic and preclinical research. Among the different forms of immunotherapy, immune checkpoint blockade (ICB) has achieved significant effects in inhibiting the malignant progression of tumors, including digestive system tumors. But, results from clinical trials show that only a selection of tumor patients respond well to ICB (20). The difference and complexity of microenvironmental components may partially explain the heterogeneity of the ICB response among tumor patients (18). It is urgent to re-cluster digestive system tumors according to individual trait of microenvironment composition, and profile relevant clinical transformation significance in corresponding cluster.

In this study, we quantified ten major non-tumor cells and evaluated the clinical value of corresponding cell components in individual cancers, where we found that some cell components are often accompanied with poor prognosis, such as neutrophils, fibroblasts, and endothelial cells. Subsequently, we re-clustered patients with digestive system tumors based on microenvironmental components and conducted in-depth analysis including clinical prognostic difference, genomic’s level, enriched biological processes and microenvironmental component characteristics. In addition, we found that the stromal score robustly enhanced in cluster 3 subgroup, which was consistently correlated with multiple negative immune cell components. We proposed that the inhibitory immune status may be characterized by high stromal scores. In the IMvigor210 database, the response rate of ICB immunotherapy for patients with high stromal scores was significantly limited, which confirmed the relationship between stromal components and the inhibitory immune microenvironment.

## Materials and Methods

### Data Acquisition

The normalized RNA sequencing and clinical information of 1,526 patients were downloaded from the UCSC website (https://genome.ucsc.edu/). For genomic level analyses, we downloaded these six types of tumors’ mutation data (MAF file) from https://portal.gdc.cancer.gov/ and Firehose (http://gdac.broadinstitute.org/). Two immunotherapy cohorts were the IMvigor210 cohort and GSE78220 respectively; the former was downloaded from http://research-pub.Gene.com/IMvigor210CoreBiologies, and the latter was obtained from https://www.ncbi.nlm.nih.gov/geo/query/acc.cgi?acc=GSE78220 (21, 22).

### The Quantification of Microenvironment Components and K-Means Clustering Analysis

The ESTIMATE R package was used to calculate stromal and immune scores, and tumor purity was calculated according to the formula from Yoshihara and colleagues (23). The relative immune cell proportions were calculated based on the CIBERSORT algorithm (24). MCP counter was conducted to calculate the enrichment of several critical immune and stromal cell components (25). The cluster analyses based on the MCP counter results were performed by consensus unsupervised analysis according to the ClusterProfiler R package, which was used to identify the most proper category from the scale of microenvironment components (26).

### Differential Enriched Biological Process and Driver Mutations

Limma R package was used to calculate the differentially expressed genes among different groups. We quantified tumor related biological process by using Gene set variation analysis (GSVA), which was further conducted to explore differential signaling pathways among different groups (27). Maftools R package and Pheatmap package were performed to illustrate significant differential driver mutations between different groups.

### Statistical Analysis

R 3.6.1 (https://www.r-project.org/) and GraphPad Prism 7 were used for statistical analysis. Kaplan–Meier survival analysis was used to evaluate the prognostic value. The receiver operating characteristic (ROC) curve was made using GraphPad Prism 7 software. A Student’s t-test was performed to analyze differential expressed expression. Chi-square test is used to evaluate the difference in treatment response between two groups. Survival curves were exported by GraphPad Prism 7. Two-tail p-value <0.05 was termed as significant.

## Results

### Overall Profiling of the Digestive System Tumor Microenvironment Components and Their Clinical Value

**Figure 1:**
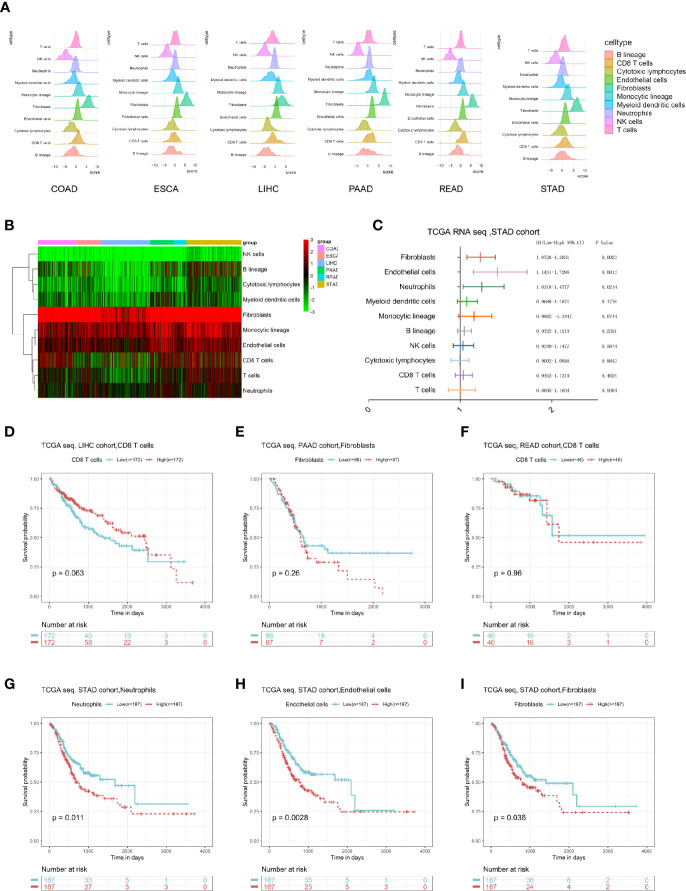
Overall description of the digestive system tumor microenvironment components and clinical value. **(A)** The landscape of microenvironmental components in digestive system cancers. **(B)** A heatmap of the MCP-counter results in these six cancers. **(C)** Univariate Cox results of each cell component in the STAD cohort. **(D–I)** The log-rank survival curve of some type of cells in specific cancer with prognostic value.

We first calculated the microenvironmental components of each digestive system of tumor patient through the MCP counter package and performed a visual exhibition (**Figure 1A**); we found that the content of fibroblasts is the most enriched, and the content of NK cells is less enriched. Similarly, by drawing a heatmap, we can clearly see that in the microenvironment of digestive system tumors, stromal components like fibroblast and endothelial cells were more enriched in environment, where monocytes are the main immune component (**Figure 1B**). Next, we calculated the impact of each microenvironment component on the survival prognosis of tumor patients through univariate Cox regression analysis in each type of tumor. There were several survival-related microenvironment component of stomach adenocarcinoma (STAD) (**Figure 1C**). We used the log-rank test method to draw survival curves of the microenvironment components, aiming to identify survival-related components in different tumors. We found that the survival-related microenvironment components in liver hepatocellular carcinoma (LIHC), pancreatic adenocarcinoma (PAAD), and rectum adenocarcinoma (READ) are not well predicted under the log-rank test (**Figures 1D–F**). While in STAD, Cox results suggested that neutrophils, fibroblasts, and endothelial cells are associated with poor prognosis, which were also significantly related to the prognosis under the log-rank test (**Figures 1G–I**).

### Re-Clustering Patients With Digestive System Tumors Based on Microenvironmental Components

**Figure 2:**
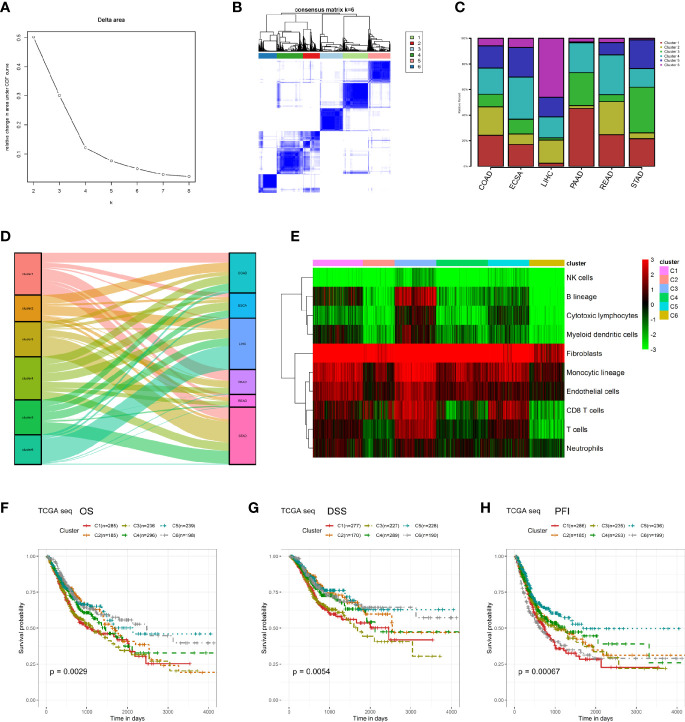
Re-clustering digestive system tumor patients based on microenvironmental components. **(A, B)** K means unsupervised clustering of patients with digestive system tumors based on the characteristics of their microenvironmental components. **(C)** The proportions of the six kinds of clusters in each type of digestive system tumor. **(D)** Sankey diagram was performed to depict the correspondence between cancer species and clusters. **(E)** The heatmap of the microenvironmental components in these six clusters. **(F–H)** Survival analyses between these six clusters in OS, DSS, and PFI.

We performed K means unsupervised clustering of patients with digestive system tumors based on the characteristics of the microenvironmental components calculated by MCP (**Figures 2A, B**), and the results showed that the six types of discrimination were the best. We analyzed the proportions of the six categories in each type of digestive system tumor. The results showed that the COAD and READ had a relative average distribution among these six clusters, while cluster 4 in ESCA was relatively enriched and cluster 6 in LIHC was the main component. Cluster 1 was dominant in PAAD, and cluster 3 had the highest proportion in STAD (**Figure 2C**). We further performed a Sankey diagram to depict the correspondence between cancer species and clusters (**Figure 2D**). Moreover, we described the main non-tumor cell components in different clusters and found that cluster 3 contains the highest content of immune and stromal cells, while cluster 6 has relatively low numbers of immune and stromal cells (**Figure 2E**). Survival analysis suggests that cluster 1 and cluster 3 have a relatively poor prognosis, where both have a high proportion of microenvironmental components, while cluster 6 has a relatively good prognosis, displaying a low proportion of non-tumor cells (**Figures 2F–H**).

### Profiling of Cluster-Related Mutations at the Genome Level

**Figure 3:**
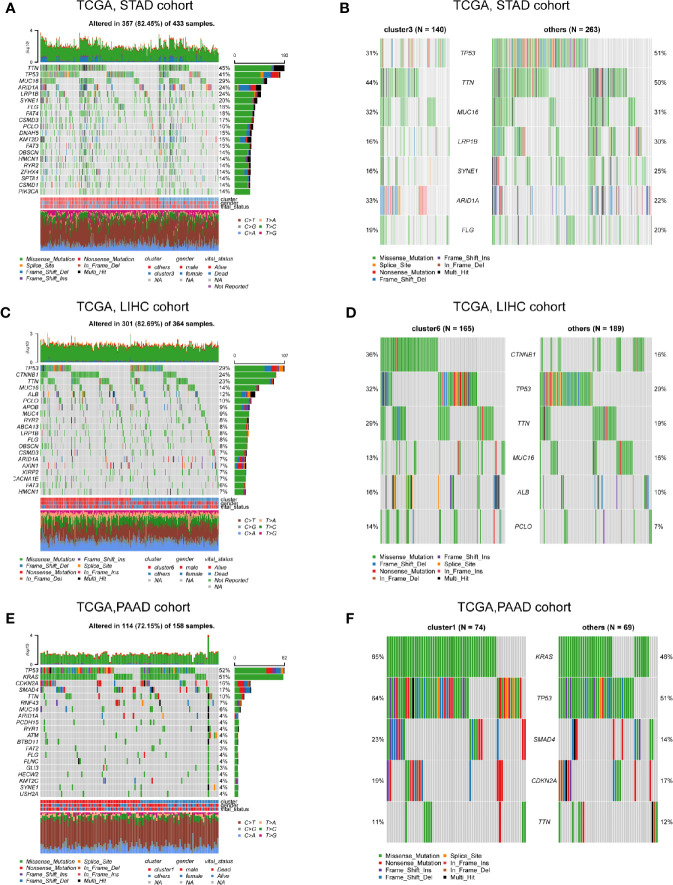
Profiling of cluster-related mutations at the genome level. **(A)** The landscape of classical driver gene mutations in the C3 and non-C3 clusters of the STAD cohort. **(B)** The most different driver mutations between C3 and non-C3 clusters of STAD cohort. **(C)** The landscape of classical driver gene mutations in the C6 and non-C6 clusters of the LIHC cohort. **(D)** The most different driver mutations between C6 and non-C6 clusters of the LIHC cohort. **(E)** The landscape of classical driver gene mutations in the C1 and non-C1 clusters of the PAAD cohort. **(F)** The most different driver mutations between C1 and non-C1 clusters of the PAAD cohort.

In order to compare the differences between different clusters at the genome level, we obtained the Single Nucleotide Polymorphisms (SNP) mutation data of these six digestive system tumors. Since the cluster 3 subgroup is accompanied by a high level of non-tumor microenvironment components and tends to be distributed in STAD, we have analyzed the classic tumor driver gene mutations in the cluster 3 and non-cluster 3 subgroups of STAD to exclude the influence of the tumor type. The results showed that the cluster 3 subgroup of STAD patients was accompanied by a low TP53 mutation rate and a high LRP1B mutation rate, suggesting a potential upstream mechanism for the poor prognosis and increased infiltration of non-tumor components of cluster 3 (**Figures 3A, B**). Similarly, due to the relatively large proportion of cluster 6 in LIHC, we analyzed the classic driver gene mutations of cluster 6 and non-cluster 6 subgroups of LIHC patients. We found that the cluster 6 subgroups of LIHC patients were accompanied by higher CTNNB1 and TTN mutations (**Figures 3C, D**). As PAAD occupied a large proportion of cluster 1, we compared the genomic differences between the cluster 1 subgroup and the non-cluster 1 subgroup, we found that the C1 subgroup of PAAD was accompanied by a higher mutation rate of KRAS and SMAD4, suggesting a potential mechanism for the poor prognosis of cluster 1 patients (**Figures 3E, F**).

### Differential Function Enrichment Analysis Among Clusters

**Figure 4:**
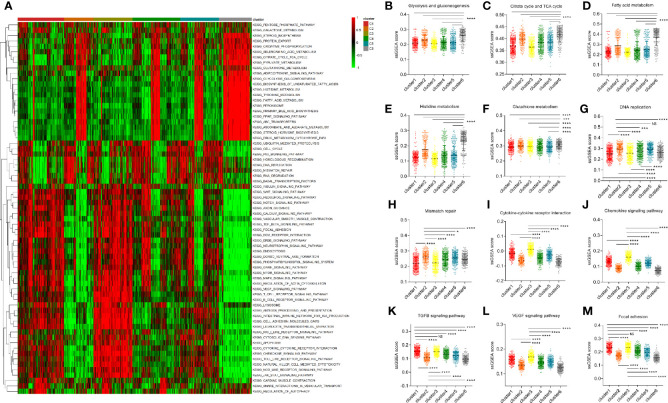
Differential function enrichment analysis among clusters. **(A)** More than 70 classical tumor-related pathways were quantified by ssGSEA method and exhibited in a heatmap. **(B–F)** Several metabolic pathways enhanced in cluster 6. **(G, H)** The level of DNA replication and mismatch repair of cluster 2 is significantly higher than that of other subgroups. **(I–M)** Cluster 3 was enriched with immune microenvironmental related terms such as the interaction of cytokines and receptors, chemokines, TGFB pathway, VEGF pathway, and focal adhesion pathway. (NS means no significance, * means P < 0.05, *** means P < 0.001, **** means P < 0.0001).

In order to explore the underlying mechanism of differences in clinical and survival characteristics of patients in different clusters, we selected more than 70 classical tumor-related pathways or critical biological processes and calculated the corresponding ssGSEA score for each tumor patient. Then, we displayed the results using a heatmap and found that some pathways that regulate the malignant behavior of tumor cells and immune-related pathways are significantly enriched in cluster 3, which is characterized by a high infiltration of non-tumor cells (**Figure 4A**). Subsequently, we conducted a series of comparisons of cancer hallmarks. In terms of several classical metabolic pathways like glucose and lipid metabolism, *etc*. The enrichment of cluster 6 was significantly higher than that of other clusters. This may be because cluster 6 is mainly composed of tumor cells (**Figures 4B–F**). The level of DNA replication and mismatch repair of cluster 2 was significantly higher than that of the other clusters (**Figures 4G, H**). Cluster 3 focused on the interaction of cytokines and receptors, chemokines, TGF*β* pathway, VEGF pathway, and focal adhesion pathway, which further suggested that microenvironmental factors may lead to the unique clinical characteristics of cluster 3 patients (**Figures 4I–M**).

### Cluster 3 Is Closely Related to the Characteristics of an Immunosuppressive Microenvironment

**Figure 5:**
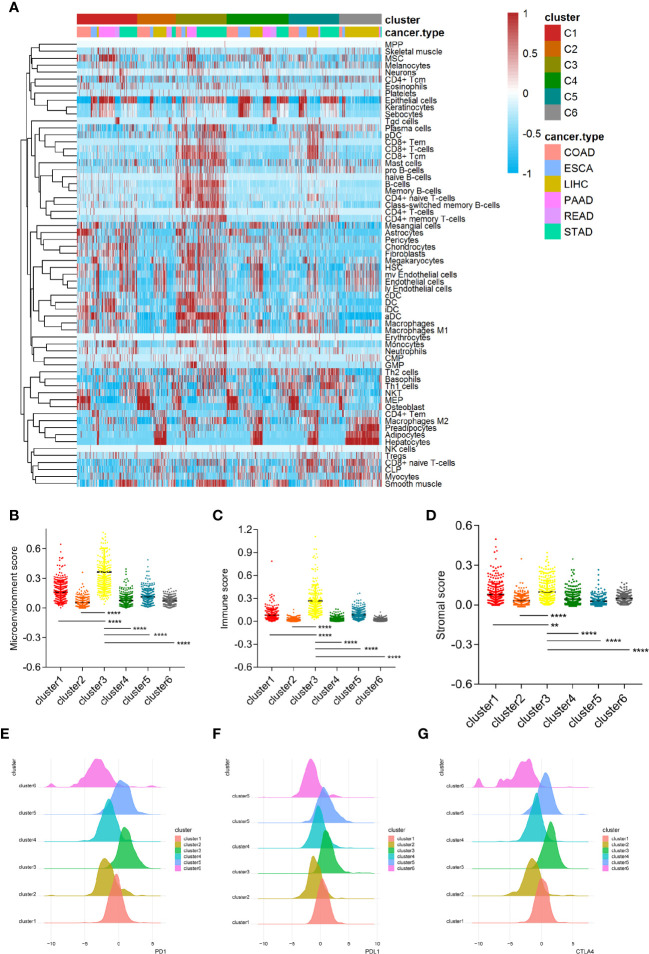
Cluster 3 is closely related to the characteristics of the immunosuppressive microenvironment. **(A)** The microenvironmental components of the six clusters conducted by X cell method. **(B–D)** The microenvironment score, immune score and stromal score in cluster 3 are significantly higher than other subgroups. **(E–G)** Classical immune checkpoints such as PD1, PDL1, and CTLA4 in cluster 3 and cluster 5 subgroups were significantly increased. (** means P < 0.01, **** means P < 0.0001).

In order to further evaluate the microenvironment characteristics of patients in cluster 3, we performed X-cell analysis and displayed the results of each cluster subgroup using a heatmap. The results showed that the immune cell and stromal cell components in cluster 3 were robustly enriched, including both activated and suppressed immune cell components (**Figure 5A**). Moreover, the microenvironmental, immune and stromal scores in cluster 3 were significantly higher than other clusters (**Figures 5B–D**). As for the classical inhibitory immune checkpoints, we found that PD1, PDL1, and CTLA4 molecules in cluster 3 and cluster 5 were significantly increased compared to other clusters (**Figures 5E–G**).

### Stromal Score Could Be Used to Predict the Response of Anti-PD1/PDL1 Treatment

**Figure 6:**
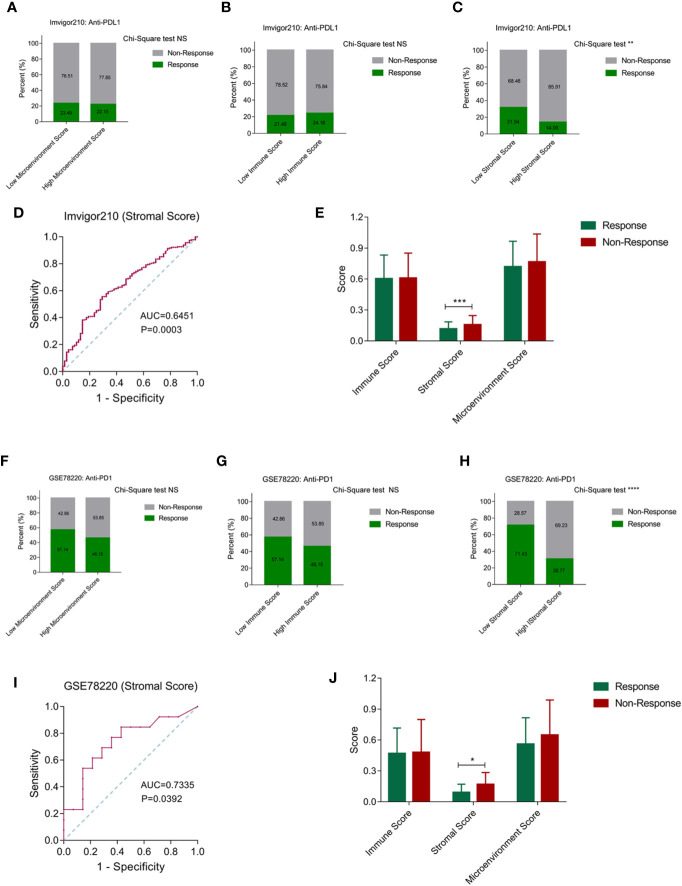
Stromal score can be used to predict the effect of anti-PD1/PDL1 treatment. **(A, B)** Patients were stratified by the microenvironment score and immune score, while there is no significant difference in the immunotherapy response rate between the high and low score groups. **(C)** The immunotherapy response rate in the high stromal score group was significantly lower than that in the low stromal score group. **(D)** The ROC curve showed that the stromal score had a well predictive effect on the positive response rate. **(E)** The stromal score in the positive response group was significantly lower than non-response group. **(F–H)** Among microenvironmental score, immune score and stromal score, only stromal score has the value in distinguishing patients with positive treatment response in GSE78220 cohort. **(I)** The ROC curve showed that the stromal score had a well predictive effect on the positive response rate in GSE78220 cohort. **(J)** The stromal score in the positive response group was significantly lower than non-response group in GSE78220 cohort. (NS means Chi-Square test no significance, * means P < 0.05, ** means P < 0.01, *** means P < 0.001, **** means P < 0.0001).

Based on the robustly enhanced microenvironmental components and suppressive immune status of cluster 3, we proposed that tumor patients with cluster 3 traits may be insensitive to immunotherapy. We represented the characteristics of cluster 3 by microenvironment, immune and stromal scores, and then evaluated the effective response rate of different groups in the ICB immunotherapy cohorts. In the Imvigor210 anti-PDL1 immunotherapy cohort, we found that there is no significant difference in the immunotherapy response rate between the high and low score groups when the microenvironment score and immune score were used as the stratified criteria (**Figures 6A, B**). When the stromal score was used as the distinction criteria, we found that the immunotherapy response rate in the high score group was significantly lower than that in the low scoring group (**Figure 6C**). Moreover, the ROC curve showed that the stromal score had a predictive effect on the positive response rate (**Figure 6D**). In addition, the stromal score in the response group was also significantly lower than in the no response group (**Figure 6E**). Similarly, we observed a similar conclusion in the GSE78220 anti-PD1 immunotherapy cohort, where the stromal score is more effective in distinguishing patients with positive treatment response than the microenvironment and immune scores (**Figures 6F–H**). In addition, the stromal score has a good predictive effect on the positive response to ICB treatment. The stromal score of the response group was significantly lower than that of unresponsive group (**Figures 6I, J**).

## Discussion

Increasing studies have shown that the microenvironmental components of malignant tumors are important factors affecting the poor prognosis and low response to treatment (28, 29). The non-tumor cell components and tumor cell components in the microenvironment can mutually regulate and transform each other to accelerate the malignant progress of tumors (16). There were a few studies that tried to enhance the immunotherapy efficiency by remodeling the microenvironment (30–32). Analyzing the microenvironment composition mode of different tumor patients can provide a certain guidance value for the selection of the next treatment strategy such as immunotherapy. Thus it is urgent to identify the subgroups of patients with different responsiveness to ICB treatment from the scale of microenvironment factors. This study first quantified eight key microenvironment components of patients with digestive system tumors and found that the two main stromal components of fibroblasts and vascular endothelial cells were prefer enriched, and the most enriched immune components were monocytes. We further evaluated the clinical value of these key microenvironmental components and found that fibroblasts, vascular endothelial cells, and neutrophils are closely related to poor prognosis.

While classification of digestive system tumors is mainly based on clinical parameters, such as tissue source, TNM stage, and grade, this study clustered patients based on characteristics of their microenvironment composition. We believe that tumors that share similar microenvironment compositions may have similar clinical characteristics. Based on the characteristics of the microenvironmental components calculated by MCP-Counter, we performed K means unsupervised clustering on patients with digestive system tumors and divided these patients into six clusters. We quantified the main non-tumor cell components in different clusters and found that cluster 3 has the highest content of immune and stromal cells, and cluster 6 has a relatively low content of immune and stromal cells. Survival analysis suggested that the prognosis of cluster 1 and cluster 3 was relatively poor, which have a high proportion of microenvironmental components, while cluster 6 has a lower proportion of non-tumor cells but exhibits a relatively good prognosis.

In addition, in order to explore the underlying mechanism of the differences between patients in different clusters, we quantified classical tumor driven biological processes. We found that in cluster 3, some pathways that regulate the malignant behavior of tumor cells and immune-related pathways were significantly enriched. In addition, cluster 3 was significantly enriched in microenvironment-related functions, such as cytokine–receptor interactions, chemokines, TGFB pathway, VEGF pathway, and focal adhesion pathway; those were all involved with microenvironment remodeling and could be termed as targeted signaling in the immunotherapy (33–37), which further suggested that microenvironmental factors may contribute to the unique clinical features of cluster 3. Based on the significantly enriched microenvironmental components and suppressive immune status of cluster 3, we speculate that tumor patients with cluster 3 characteristics may be insensitive to immunotherapy. We replaced the characteristics of cluster 3 with microenvironmental score, immune score and stromal score respectively, and then tested the treatment response rate of different groups in the ICB immunotherapy cohorts. First of all, in the Imvigor210 anti-PDL1 immunotherapy cohort, we found that when the stromal score was used as the distinguishing standard, the immunotherapy response rate in the high score group was significantly lower than that in the low score group (22). The ROC curve showed that the stromal score had a good predictive effect on the positive response rate. The stromal score in the positive response group was also significantly lower than that in the non-response group. We also reached a similar conclusion in the GSE78220 anti-PD1 immunotherapy cohort (21), where stromal score was more effective in distinguishing patients with positive response to ICB treatment.

In summary, the composition of the microenvironmental components of various tumors in the digestive system is heterogeneous. There is a subgroup of patients characterized with high stromal and immune components that are accompanied with a poor prognosis and insensitivity to ICB therapy. Our research provides a new approach for precise diagnosis and treatment of digestive system tumor patients.

## Data Availability Statement

Publicly available datasets were analyzed in this study. These data can be found here: http://genome.ucsc.edu/, https://portal.gdc.cancer.gov/, http://gdac.broadinstitute.org/, http://research-pub.Gene.com/IMvigor210CoreBiologies, https://www.ncbi.nlm.nih.gov/geo/query/acc.cgi?acc=GSE78220.

## Author Contributions

WY and GY conceptualized and designed the study. BB and WS downloaded the data and contributed to the data curation. CZ developed the methodology. WY and GS analyzed and interpreted the data. WY and GY wrote and revised the manuscript. All authors contributed to the article and approved the submitted version.

## Funding

This work was supported by the Science and Technology Research Project for Colleges and Universities in Hebei Province (grant number: QN2016060, QN2015044), Launch Fund for High-Level Talents Scientific Research of Chengde Medical College (grant number: 202002), and Medical Scientific Research Project for Hebei Provincial Health and Family Planning Commission (grant number: 20160313, 20160310).

## Conflict of Interest

The authors declare that the research was conducted in the absence of any commercial or financial relationships that could be construed as a potential conflict of interest.

## Abbreviations

ICB, Immune checkpoint blockade; TCGA, The Cancer Genome Atlas; SCNA, Somatic copy number variation; GSVA, Gene set variation analysis; ROC, Receiver operating characteristic; STAD, Stomach adenocarcinoma; LIHC, Liver hepatocellular carcinoma; PAAD, Pancreatic adenocarcinoma; READ, Rectum adenocarcinoma; COAD, Colon adenocarcinoma; ESCA, Esophageal adenocarcinoma; SNP, Single Nucleotide Polymorphisms.
